# Cannabidiol (CBD) Alters the Functionality of Neutrophils (PMN). Implications in the Refractory Epilepsy Treatment

**DOI:** 10.3390/ph14030220

**Published:** 2021-03-05

**Authors:** Claudia Taborda Gómez, Fabiana Lairion, Marisa Repetto, Miren Ettcheto, Amalia Merelli, Alberto Lazarowski, Jerónimo Auzmendi

**Affiliations:** 1 Departamento de Bioquímica Clínica, Facultad de Farmacia y Bioquímica, Instituto de Fisiopatología y Bioquímica Clínica (INFIBIOC), Universidad de Buenos Aires, Buenos Aires C1120AAF, Argentina; clatamago@gmail.com (C.T.G.); amerelli2002@yahoo.com.ar (A.M.); nadiatom@ffyb.uba.ar (A.L.); 2 Departamento de Química Analítica y Fisicoquímica, Facultad de Farmacia y Bioquímica, Instituto de Bioquímica y Medicina Molecular, Consejo Nacional de Investigaciones Científicas y Técnicas (CONICET), Universidad de Buenos Aires, (IBIMOL, UBA-CONICET), Buenos Aires C1113AAD, Argentina; flairion@ffyb.uba.ar (F.L.); mrepetto@ffyb.uba.ar (M.R.); 3 Department of Pharmacology, Toxicology and Therapeutic Chemistry, Faculty of Pharmacy and Food Science, Institute of Neuroscience, University of Barcelona, 08193 Barcelona, Spain; mirenettcheto@ub.edu; 4 Biomedical Research Networking Centre in Neurodegenerative Diseases (CIBERNED), 28031 Madrid, Spain; 5 Consejo Nacional de Investigaciones Científicas y Técnicas (CONICET), Buenos Aires C1425FQD, Argentina

**Keywords:** cannabidiol, refractory epilepsy, polymorphonuclear neutrophils (PMNs), chemotaxis, oxygen consumption, oxidative stress

## Abstract

Cannabidiol (CBD), a lipophilic cannabinoid compound without psychoactive effects, has emerged as adjuvant of anti-epileptic drugs (AEDs) in the treatment of refractory epilepsy (RE), decreasing the severity and/or frequency of seizures. CBD is considered a multitarget drug that could act throughout the canonical endocannabinoid receptors (CB1-CB2) or multiple non-canonical pathways. Despite the fact that the CBD mechanism in RE is still unknown, experiments carried out in our laboratory showed that CBD has an inhibitory role on P-glycoprotein excretory function, highly related to RE. Since CB2 is expressed mainly in the immune cells, we hypothesized that CBD treatment could alter the activity of polymorphonuclear neutrophils (PMNs) in a similar way that it does with microglia/macrophages and others circulating leukocytes. In vitro, CBD induced PMN cytoplasmatic vacuolization and proapoptotic nuclear condensation, associated with a significantly decreased viability in a concentration-dependent manner, while low CBD concentration decreased PMN viability in a time-dependent manner. At a functional level, CBD reduced the chemotaxis and oxygen consumption of PMNs related with superoxide anion production, while the singlet oxygen level was increased suggesting oxidative stress damage. These results are in line with the well-known CBD anti-inflammatory effect and support a potential immunosuppressor role on PMNs that could promote an eventual defenseless state during chronic treatment with CBD in RE.

## 1. Introduction

Epilepsy is a neurological disease affecting about 50 million people worldwide, and nearly 30–40% of patients will develop a multidrug-resistant (MDR) phenotype defined as refractory epilepsy (RE), characterized by high recurrence of seizures that cannot be controlled by at least two well-tolerated antiepileptic drugs (AEDs) appropriate for the particular epilepsy type [1,2,3]. Despite the constant develop of the AEDs with several improves in absorption, tolerance, molecular target and diminished side effects, the rate of RE remains unchanged through the decades. Several mechanisms have been described that explain this phenotype, including the role of ATP binding cassette (ABC)-transporters, particularly P-glycoprotein (P-gp) [4,5].

Several alternative therapies such as ketogenic diet and vagal stimulation have been applied with variable results. In this context, the treatment with cannabinoids compounds such as cannabidiol (CBD) have emerged as a promising adjuvant therapy for seizure control [6]. Several reports showed a decrease in the seizures frequency and intensity [7,8,9] and that correlates with an increased plasmatic AEDs concentration [10,11,12]. Despite the beneficial effect of CBD, only Epidiolex^®^ was approved by the Food and Drug Administration, and their action mechanism is still unknown [13]. Several lines of evidence highlight CBD as a multitarget drug that could act throughout the endocannabinoid receptors (CB1 in the central nervous system and CB2 in immune cells) as an inverse agonist [14,15] as well as throughout of others metabotropic receptors such as serotonin receptors (5-HT1A and 5-HT2A) [16], adenosine receptors (A1 and A2) [17] and G protein-coupled receptor 55 (GPR55) [16]. CBD has also been demonstrated to regulate current of Ca interacting TRPV1 [18,19] or voltage-gated T-type calcium channels (VGCC) [20]. Furthermore, CBD is able to inhibit the activity of several cytochromes responsible for AEDs metabolization. In this way, CBD inhibits the activity of CYP2C19, CYP2D6 and CYP2C9 that metabolize Phenytoin and Carbamazepine between others [21,22]. Recently, it was demonstrated that CBD has an inhibitory action on the excretory function of P-glycoprotein, highly related to drug-resistant epilepsies [5,23]. So, the chronic use of CBD in addition to common anti-epileptic drugs (AEDs) could help in the control of severity and/or frequency of seizures in patients with RE supporting the CBD adjuvant role.

The endocannabinoid system can regulate the immune response through the stimulation of CB2 receptors, which is mainly expressed in most of the immune cells including the neutrophils polymorphonuclear cells (PMNs) [24]. PMNs are produced in large numbers in the bone marrow (~10^11^ cell per day), and they represent the more abundant leukocytes in peripheral blood and are renewed from circulation three times a day [25]. The stimulation of PMN´s CB receptors with the endogenous agonist 2-arachidonoylglycerol usually leads to an anti-inflammatory effect [26]. In a rat uveitis model, the stimulation of CB2 decreased leukocyte adhesion in the iris microcirculation, inhibited the release of pro-inflammatory cytokines and chemokines and increased iridial microcirculation blood flow [27,28]. Furthermore, the in vivo and in vitro stimulations of CB2 have been related with a decreased release of metalloproteases from PMNs [29]. In the same way, the lack of CB2 increased the PMN recruitment in the CB2 knockout (CB2-KO) mice endotoxemia model [30]. Additionally, the level of the intestinal lumen in these CB2-KO mice, a decreased *N*-acyl ethanolamine-type endocannabinoids release allowed an increased PMN transmigration that correlated with the ablation of CB2 [31].

Cellular immunity activity of PMNs needs initial processes for neutrophils activation, triggering an ordered sequence of events as rolling and adhesion to activated endothelium; migration into the affected tissues by chemotaxis; recognition and binding to microorganisms; engulfment and phagocytosis of them and occasionally neutrophil extracellular tramps (NETs) production. All these processes are orchestrated by a complex tangle of cytokines that control and drive the PMNs activity [32]. The PMNs transmigration occurring prominently at the borders of endothelial cells, secondary to signals delivered by “end target-derived” chemoattractants, such as formyl peptides, released by bacteria or by mitochondria from dying cells. These are dominant signals that override “regulatory cell-derived”, such as bioactive peptides (LTB4) or chemokines (IL-8) [33]. When the PMNs arrive at the site of infection and recognize the pathogens, they activate the NADPH (nicotinamide adenine dinucleotide phosphate-oxidase/Nox2) oxidase complex to produce reactive oxygen species (ROS) with which to attack the microorganisms at the extracellular level or in phagosomes. After assembly, the NADPH oxidase complex transfer electrons from NADPH to molecular oxygen generating superoxide anions (O_2_^−^) as the primary product. To minimize damage, cells are equipped with antioxidant scavenging enzymes, such as superoxide dismutase (SOD) and catalase. SOD, which dismutates O_2_^−^ to hydrogen peroxide (H_2_O_2_) and glutathione peroxidase, can further convert these species into water, which limit damages to the host. Granule-localized myeloperoxidase (MPO) can convert H_2_O_2_ to hypochlorous acid (HOCl), which can enhance clearance of invading pathogens. MPO can also directly convert O_2_^−^ into singlet oxygen (^1^O_2_^*^). In addition, ferric iron can convert O_2_^−^ and H_2_O_2_ into hydroxyl radical (HO^•^) [34,35].

Cannabinoids compounds can also facilitate different infections supporting an immunosuppressive role [36]. Additionally, it was reported that activation of CB2 blocks monocyte migration, dampens LPS induced secretion of the pro-inflammatory cytokine tumor necrosis-alpha (TNF-α) and can to inhibits the differentiation of human monocytes into antigen-presenting dendritic cells [37], altering the inflammatory responses to infection [38]. Furthermore, it was described that CBD can induce apoptosis in human monocytes by mitochondrial oxidative stress [39]. In the same way, similar effects on microglia proliferation, migration and inflammatory response were elicited by CBD [40,41,42]. In light of these evidences, we hypothesized that CBD treatment could alter the activity of PMNs in a similar way that it does with microglia/macrophages and others circulating leukocytes leading to reduce the immunocompetence of PMNs, by producing functional alterations and/or affecting their viability.

## 2. Results

### 2.1. Effect of CBD on PMN Viability

**Figure 1:**
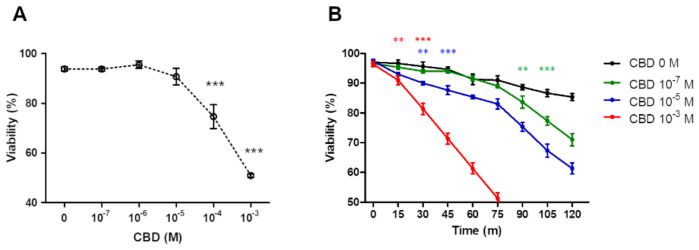
CBD affect the viability of PMNs. PMNs viability is significantly affected with CBD concentrations >10^−5^ M (**A**) and correlated with shorter survival time courses at the same CBD concentrations (**B**). Results are expressed as mean ± SD (*p* < 0.05). ** and *** indicate the significance differences relative to 0 M of CBD. In (**B**) the asterisks indicate the point from which the curves for each CBD concentration are different from the control, while the color of the asterisks indicates which CBD concentration is compared.

Under the hypothesis of the anti-inflammatory effect of CBD, argued about an increased PMN mortality, we tested the cell viability incubating 10^6^ PMN/mL with increased concentrations of CBD (10^−7^ M to 10^−3^ M). After 1 h of incubation, the micromolar CBD range did not produced any change in PMN viability, while a substantially decreased viability (77 ± 3.1%) at submilimolar CBD concentration was observed, while approximately 50% of viability was reached with millimolar CBD concentration (Figure 1A). Next, the time course of PMNs viability at low, middle and high CBD concentrations was analyzed. When the PMNs were incubated with DMSO as vehicle (DMSO was added at same concentration as CBD a 10^−3^ M) a PMN’s viability of 10% at 60 min (Figure 1B, black line) was observed. Similar results were observed with CBD 10^−7^ M, but that fall accelerated at 90–120 min. In contrast, intermediate and very pronounced loss of viability were observed with CBD at 10^−5^ M and 10^−3^ M, respectively (Figure 1B).

### 2.2. Morphological Analysis

**Figure 2:**
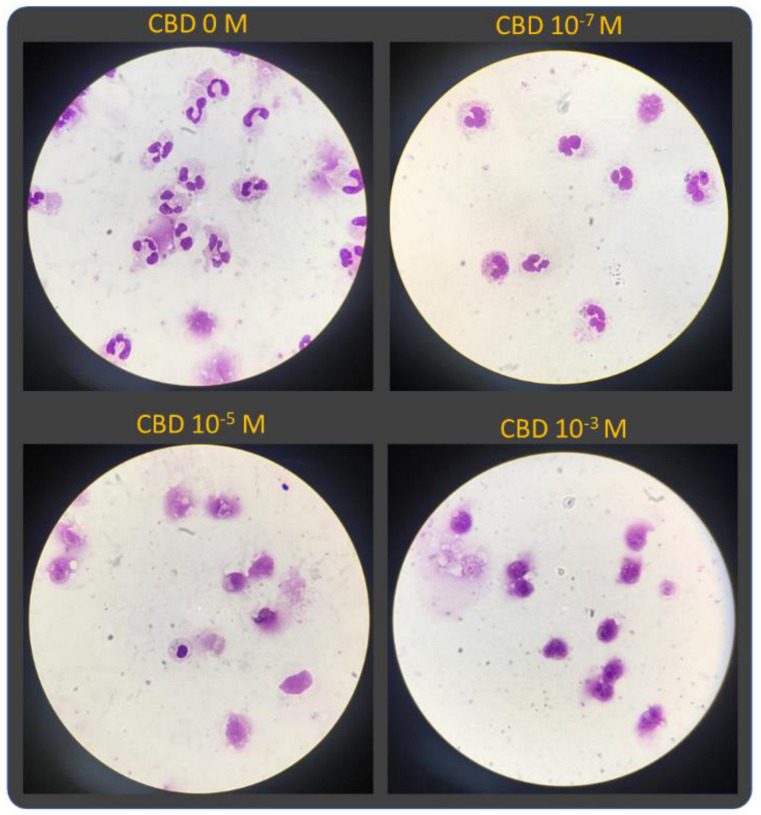
The morphology of PMNs is modified by CBD in a concentration-dependent manner. All images were captured with a magnification of 40×.

The morphological analysis revealed that PMN exposed at lower CBD concentrations did not show any significant morphological alteration compared to normal neutrophils. However, the higher concentration of CBD gradually increased the morphological alterations characterized by cytoplasmic vacuolization, nuclei with pseudo-maturation aspects, degranulation and rupture of the cytoplasmic membrane (Figure 2).

**Figure 3:**
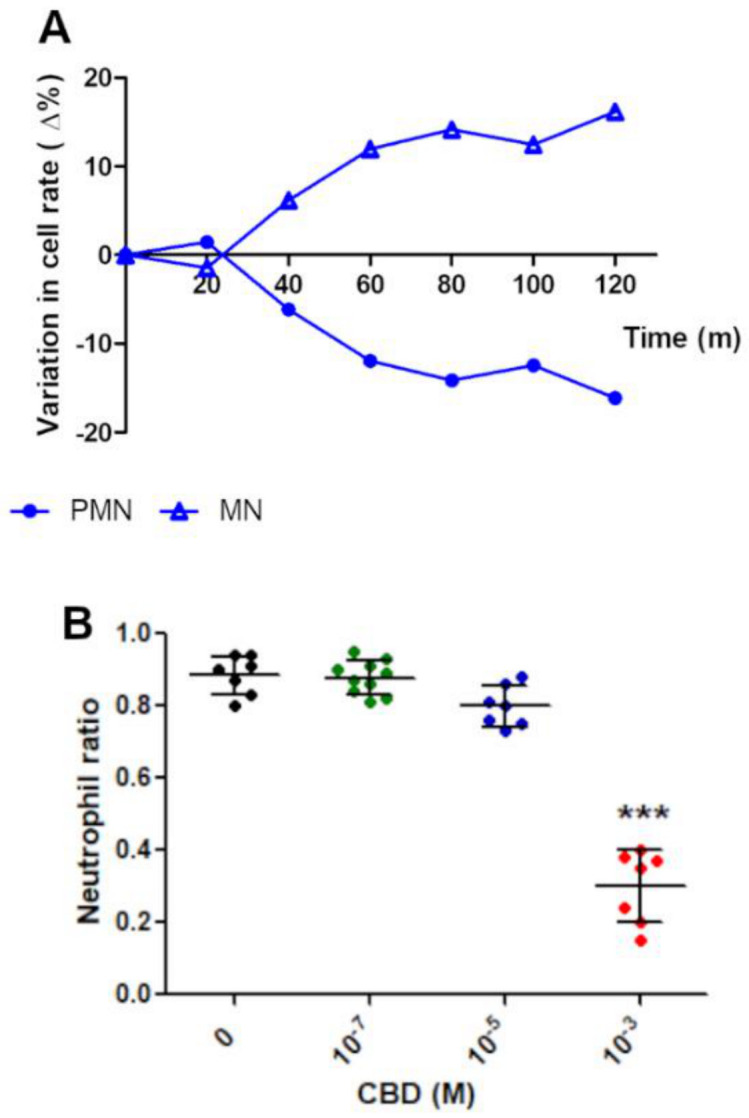
CBD accelerates the decrease in PMN/MN rate. (**A**) Spontaneous nuclear transformation from PMN to MN forms affects no more than 20% of normal PMN population in a symmetric mode. (**B**) A significant drop in the PMN/MN ratio is observed when PMNs are exposed to higher CBD concentrations. Results are expressed as mean ± SD (*p* < 0.0001). *** indicate the significance differences relative to 0 M of CBD.

In normal conditions, a spontaneous nuclear modification can be observed across the time, where normal polymorphonuclear (PMN) neutrophils’ population decreases proportionally to the increasing of abnormal mononuclear (MN) forms’ population (Figure 3A). The PMN/MN rate indicated that Figure 3B showed that the PMN/MN rate decreases significantly after one hour incubation only with higher CBD concentrations (10^−3^ M) (Figure 3).

### 2.3. Effect of CBD on PMN Functionality

**Figure 4:**
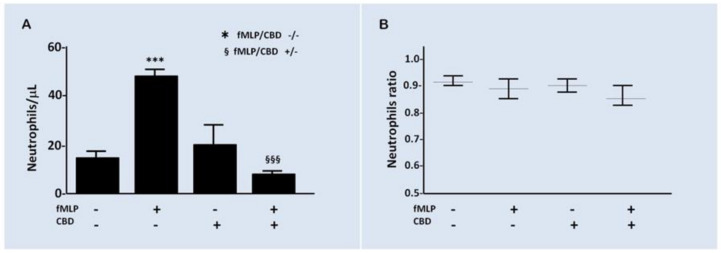
CBD modifies the PMN chemotaxis. (**A**) The activation of chemotaxis by fMLP is significantly inhibited by CBD (*p* < 0.01). *** compared to fMLP/CBD -/- while §§§ compared to fMLP/CBD +/-. (**B**) During the time of the experiment, no differences were observed in the ratio PMN/MN, indicating that chemotaxis results were independent of morphologic modifications. Results are expressed as mean ± SD (*p* = 0.1865).

As mentioned above, 10^−7^ M CBD did not produce a significant effect on the viability to short times, and then, we use this concentration to test the effect of CBD on the PMN chemotaxis elicited by fMLP 10^−7^ M using a Boyden chamber. After an incubation of 45 min, the chemotaxis induced by fMLP was increased by three times over the spontaneous basal level. On the other hand, CBD reduced the induced chemotaxis in comparison to vehicle (Figure 4A). Additionally, the PMN/MN ratio in the upper well of Boyden chamber was measured (Figure 4B) to discard if any differences of PMN/MN ratio could be related with any altered chemotaxis results. No statistical differences were observed in the PMN/MN ratio between CBD and vehicle.

**Figure 5:**
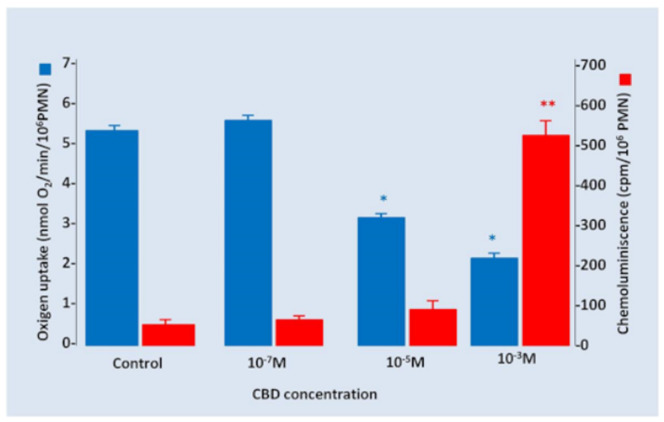
Oxygen uptake and chemiluminescence under several CBD concentrations. An inverse effect between oxygen uptake and chemiluminescence was observed secondary to the incubations of PMNs with increased concentrations of CBD. Results are expressed as mean ± SD (*p* < 0.05). * and ** indicate the significance differences relative to 0 M of CBD.

Additionally, the PMN oxygen consumption and chemiluminescence under CBD gradually increased concentrations were measured. We found that CBD 10^−7^ M had no effects on oxygen uptake by PMNs; however, a significant decrease in oxygen consumption was observed, in an apparent inverse relationship with the increase in CBD concentrations. Inversely, chemiluminescence was significantly increased only when PMNs were incubated with high concentrations of CBD 10^−3^ M (Figure 5).

## 3. Discussion

It was described that cannabidiol (CBD), a non-psychoactive metabolite of *Cannabis sativa*, is popularized as a potential medicinal product with anti-inflammatory, antioxidant and analgesic effects. Because PMN cells are clear protagonists of these mentioned conditions, we hypothesized that CBD could play an inhibitory effect on these properties, irrespective to the CBD actions on other tissues and systems. Some endocannabinoids or phytocannabinoids have been reported to inhibits the human neutrophils migration, but without effects on their viability [43].

In our study, the viability as well as chemotaxis were significantly reduced by CBD 10^−3^ M and 10^−7^ M (50 and 80%, respectively). Additionally, a loss in nuclear lobulation and morphology changes were observed in PMN exposed to CBD. Additionally, we report that the oxygen uptake of PMNs also was diminished, while chemiluminescence was significantly induced by CBD at 10^−3^ M. This dissociating effect on the two parameters of oxidative stress (O_2_ consumption and chemiluminescence) could be secondary to the poly-target CBD properties, acting on different receptors presents on PMNs and activating as well as inhibiting a wide spectrum of cell’s functionalities.

Structural and functional changes in PMNs membrane trigger the respiratory burst either by the uptake of microorganisms or by active agents at membrane level. This process includes activation of both multi-membrane-bound NADPH oxidase complex [44,45] and constitutive nitric oxide synthase (cNOS) in the external membrane of PMNs and myeloperoxidase enzyme in the internal granules. In response to a stimulus, PMNs consume oxygen (O_2_) to generate O_2_^−^ and H_2_O_2_. Experimentally, it was described that the activity of NOX2 is inhibited by CBD in PMNs and, consequently, the generation of O_2_^−^ and H_2_O_2_, preventing the oxidative burst in a direct form, interfering with translocation of the NOX2 subunits to the membrane, or indirectly, on signaling pathways needed for its activation [46].

In our experiments, CBD produced an inhibitory effect on O_2_^−^ and H_2_O_2_ generation; this effect was increased when PMNs were exposed to increasing CBD concentrations (40% for 1 × 10^−5^ M, *p* < 0.05 and 60% for 1 × 10^−3^ M, *p* < 0.01) (Figure 5). These results suggest that the inhibitory effect of CBD on NOX2 activity could be associated to a direct inhibitory effect on this protein or inducing structural changes in the membrane that prevent the binding of NOX2 subunits (p47^phox^ and p40^phox^) necessary for its normal function, as was described for chronic granulomatous disease (CGD) [47]. These results are in the same line with a recent report showing the decreased activity in microglial NADPH oxidase complex [48]. Potential mechanisms of NOX2 deactivation by CBD can include the dephosphorylation of p^hox^ subunits, decreased activity of guanine activating protein (GAPs) on Rac (small GTPase) proteins and dissemble of the complex or diminution of amount of FAD [35].

Nitric oxide (NO) is generated together with O_2_^−^ and H_2_O_2_ in the respiratory burst in PMNs exposed to chemoattractant agents as phorbol esters (PMA), by the activation of cNOS [49]. Carreras et al. showed that NO production is about 30% of the total oxygen consumption in PMN stimulated with PMA, and 70% of oxygen is used, simultaneously, for O_2_^−^ generated by NOX2 activity, involving protein kinase C as a common mechanism [50]. NO inhibits O_2_^−^ production by NOX2 and other potent oxidants in PMNs activated, the peroxynitrite (ONOO^−^) when NO reacts with O_2_^−^ [51]. However, the increase in spontaneous chemiluminescence by adding CBD in the highest concentration evaluated in this study would be indicating the generation of toxic for PMN and reactive end products of lipid peroxidation and proteins. This indicates the generation of the electronically excited product, ^1^O_2_^*^, in the neutrophils. The chemical reactivity of ^1^O_2_^*^ in solution is mainly due to electron configuration, higher energy and short life of this reactive oxygen species; ^1^O_2_^*^ emits light, as excited electrons fall to lower energy levels, and is the final product of lipid and protein oxidation process [52,53].

MPO needs H_2_O_2_ to induce toxic effects on microorganisms by mechanisms of halogenation or oxidation mediated mechanisms in neutrophils and is inhibited by hypoxia condition. MPO released from azurophilic granules catalyzes the synthesis of HO^•^ and ^1^O_2_^*^ from of H_2_O_2_ and O_2_^−^ reaction or generates hypochlorite anion (ClO^-^) from H_2_O_2_ and chloride anion (Cl^-^). The ClO^−^ anion may react with H_2_O_2_ in excess to generate ^1^O_2_^*^ [54]. Nevertheless, the finding that phagocytosis is associated to light emission, that NADPH oxidase system is inhibited with CBD and that myeloperoxidase is present in very high concentrations in the azurophil granules of PMN indicate that products of oxidation may be formed by PMN and lipid peroxidation may be initiated for oxidants other than HO^•^. The molecular mechanism for H_2_O_2_ production in CBD exposed PMNs are unknown, but it seems be similar to the CGD characteristics, in which the assembly of O_2_^−^ and H_2_O_2_-generating system, NOX2, is defective [55]. In neutrophils, the ROS production by activation of NADPH oxidase occurs not only at the plasma membrane but also at an intracellular location. Regardless of the ROS released out of the cell, and in order to give rise to an intracellular chemiluminescence reaction, ROS also needs access to MPO that is stored in the azurophil granules [56].

PMN-NADPH oxidase consists of at least of three cytosolic components and two subunits (cytochrome b), bound to both the plasma membrane and specific granule membranes, and activation of this enzyme could be related to the vesicular location of P-gp in neutrophils [57]. Furthermore, the neutrophil not only requires the presence of P-gp, but also the cystic fibrosis transmembrane conductance regulator (CFTR) into the phagosomal membrane to allow an increase in Cl^-^ transport into the phagosome for the support of HOCl generation [58]. Because both P-gp and CFTR are members of the ABC-transporters superfamily, the inhibitory effects of CBD on P-gp, as previously demonstrated [23] could also inhibit the CFTR activity and, in consequence, increase the risk of infection, resulting in inadequate microbial killing, as described in the cystic fibrosis disease [59,60].

Increasing clinical evidence supports the role of CBD as an adjuvant therapy for the control of refractory epileptic seizures. In this context, CBD has been shown to decrease the frequency and intensity of epileptic seizures, even keeping a variable proportion of people seizure-free [7,8,9]. Additionally, treatment with CBD showed an increase in the AEDs plasma concentration [10,11,12]. Although the mechanism of this increase is not clear, it is known that CBD blocks CYP3A and CYP2C cytochromes that are responsible for the AEDs metabolization [9]. Recently, our group has shown that CBD can block the excretory activity of P-gp in a similar way to the specific blocker Tariquidar [23]. This would possibly contribute to reducing the clearance of AEDs and also make their access to the brain parenchyma more effective, bypassing the excretory activity of P-gp in the blood–brain barrier. Several reports show that CBD has an anti-inflammatory effect by reducing the secretion of pro-inflammatory mediators and that it can produce an immunoparalysis acting on lymphocytes, monocytes, macrophages and even microglia [37,38,39,40,41,42]. However, the role of CBD on the activity of PMNs has been little investigated. In this context, we show that CBD decreases the viability and functionality of PMNs consequently with its anti-inflammatory effect. From a clinical perspective, the anti-inflammatory effect of CBD is appropriate for reducing brain inflammation that accompanies epileptic crises. However, the chronic use of CBD can cause a state of immune paralysis, responsible for a greater susceptibility to infections. Although CBD facilitates better control of seizures in patients with RE, its harmful effect on the functionality of the neutrophils shown here, and as well as on other cells of the immune system [61], may require a clinical-immunological control in patients with RE who are chronically medicated CBD.

## 4. Materials and Methods

### 4.1. Ethic Statements and Samples

Samples were collected from the Hematology Laboratory of the Clinical Biochemistry Department of Clinical Hospital “José de San Martín”. The blood samples were collected with EDTA and were only used if they presented normal results in the cell blood counter analysis. This procedure was approved by CEIC (Comité de Ética en Investigación Clínica) from Facultad de Farmacia y Bioquímica—Universidad de Buenos Aires (Exp-UBA 77388/2017).

### 4.2. Materials

Cell culture reagents were obtained from Invitrogen Life Technologies (Carlsbad, CA, USA). Giemsa solution was provided by Biopack (cod 1100.08- Biopack-Buenos Aires, Argentina). Other reagents such as Ficoll–Paque plus (GE17-1440-03), fMLP (F3506) and Trypam Blue (T6146) were obtained from Merck (Darmstadt, Germany).

### 4.3. CBD Preparation

CBD was diluted in dimethyl sulfoxide (DMSO) at a final concentration of 10^−1^ M. This stock solution was aliquoted and stored at −20 °C. For each experiment, intermedial solutions were prepared from stock solution with appropriated volume of buffer phosphate saline (PBS). A final CBD experimental concentration was reached using an appropriated volume of PMN suspension. Control experiments using DMSO as a vehicle were performed, adding an equal volume of DMSO that needed of the stock solution to reach a CBD 10^°^ M concentration.

### 4.4. PMNs Isolation

PMNs were isolated, as described previously [62]. Briefly, PMNs were isolated from blood samples using the Ficoll–Paque plus method and exposed to hypotonic shock with sterile NaCl solution (0.2% *p/v*) for 20 s and rebalanced with an equal volume of sterile NaCl solution (1.6% *p/v*). This suspension of PMNs was centrifuged 10 min at 450 g for 10 min at 20 °C, the supernatant fluid was discarded, and the pellet was resuspended in 10 mL of sterile RPMI 1640 medium at 37 °C.

In all assay, aliquots of a final PMN 10^6^/mL suspension were incubated with increased concentrations of CBD (10^−7^ M, 10^−5^ M and 10^−3^ M) at 37 °C and compared with aliquots of PMN 10^6^/mL suspension without CBD that were used as normal control.

### 4.5. PMNs Count and Viability

Total and differential cells account of viable PMNs in suspension were developed using the Sysmex XN-1000 AS-01 Automated Hematology Analyzer (XN-Series, Sysmex^®^ Chuo-ku, Kobe, Hyogo 651-0073, Japan), in a mode that allowed for distinguishing the mono-nuclear (MN) cells from neutrophils. These counts were confirmed manually with Neubauer´s chamber, using a 1/400 dilution of the PMNs suspension in Turk’s solution (1–2% acetic acid with aqueous methylene blue).

The PMNs’ viability was measured with equal volume of Trypan Blue solution 0.4% using a 1/100 dilution of the abovementioned neutrophils suspension. PMN/MN ratio as well as the cell´s viability was determined before and after each experiment, and only cells suspensions with viability >95% were used.

### 4.6. Morphological Analysis

Sample film preparations was developed using a volume of 100 µL of 10^5^ PMN/mL suspension, spread-out on glasses by cito-spinning centrifugation, fixed with methanol 10% for 10 min and stained with Giemsa. After washes with water, glasses were dried at room temperature overnight. Glasses were observed at 40× magnification and photographed using an Olympus IX-81 microscope equipped with a DP71 camera (Olympus, Tokyo, Japan).

Additionally, the transformation of poly-lobed morphology of normal PMN to mono-nuclear (MN) cells can indicate the kinetics of viability loss secondary to the incubation of PMN with CBD detected as the PMN/MN cells ratio, by Sysmex XN-1000 AS-01 Automated Hematology Analyzer. So, the PMN/MN rate can reflex the nuclear condensation index related to unviable PMN amounts.

### 4.7. Chemotaxis Assay

PMN migration assays were performed in 12 multi-wells Boyden chambers, and polyvinylpyrrolidone-free polycarbonate filters with pores 5 µm in diameter were used. The chemo-attracter *N*-formyl-Met-Leu-Phe peptide (fMLP) (10^−7^ M) and/or CBD (10^−3^ M) were carefully loaded in the lower well, avoiding the formation of air bubbles. After the chambers were assembled, upper wells were filled with the suspension of 10^6^ PMNs/mL. In the settings of the experiment, the chambers were incubated at 5% CO_2_ and 37 °C for 0, 30, 60 and 90 min, establishing that the optimal incubation time was 45 min. Finally, transmigrated cells were counted using a Sysmex XN-1000 AS-01, as described above. Additionally, the ration of PMN/MN was performed in both the upper and lower cells’ suspensions. All experiments were assayed by triplicate.

### 4.8. Stimuli and Activation of PMN

Human PMN cells (1 × 10^6^ cells) suspended in buffer phosphate solution (PBS: 138 mM NaCl, 2.7 mM KCl, 8.1 mM Na^2^HPO^4^ and 1.47 mM KH^2^PO^4^ buffer, pH 7.4) were preincubated for 15 min at 37 °C with increasing concentrations of CBD (10^−7^ M; 10^−5^ M and 10^−3^ M).

### 4.9. PMN Oxygen Consumption

A liquid-phase respiration measurement system, the oxygraph plus model, was used for the determination of oxygen consumption by PMN, consisting of an oxygen electrode chamber (DW1/AD model) with integral Clark type polarographic oxygen electrode (S1) (Hansatech Instruments Ltd., Norfolk, British), thermostated at 37 °C, with human PMN (10^6^/mL) in PBS supplemented with 0.9 mM CaCl_2_, 0.5 mM MgCl_2_ and 7.5 mM glucose (PBSG). For the assay, respiratory buffer (for 1 mL final volume) was placed in the electrode chamber. The chamber was covered. Substrates were added with a Hamilton syringe. The rate of oxygen uptake was calculated from the initial time course and expressed as nmol of oxygen/min/10^6^ cells, as previously described [49].

### 4.10. PMN Chemiluminescence

Chemiluminescence is a useful approach to determine the occurrence of oxidative stress in cells or tissues. Spontaneous chemiluminescence in fresh PMNs was assayed, applying a photon counter and the procedure for the light emission reading use, as previously described [63,64]. Results are expressed in cps/mL PMN (cps: counts per second, 1 cps corresponds to about 10^3^ photons per second).

### 4.11. Statistics

All samples for PMN viability and chemotaxis experiments were run three times, while samples for PMN chemiluminescence and oxygen consumption measurements were tested once. All experiments were performed by triplicate. After checking the normal distribution, differences were analyzed by Student *t*-test or one-way ANOVA with a Dunnett’s post-test comparison with a significance level alpha = 0.05.

## 5. Conclusions

Cannabidiol, a lipophilic cannabinoid compound without psychoactive effects, has emerged as adjuvant of AEDs in the treatment of RE. The treatment with CBD as adjuvant has been reported for decreasing the severity and/or frequency of seizures. Additionally, CBD has a well-documented anti-inflammatory role, decreasing the microglial reactivity between others. In this sense, microglia and PMN share several immune activities. The results of this work show that CBD decreases PMN survival, chemotaxis and oxygen uptake, while increasing the singlet oxygen formation, suggesting that CBD could affect the PMN functionality. In a clinical perspective, a prolonged CBD treatment could lead a defenseless state, increasing the infections risk. This side effect could be bypassed with an antioxidants treatment, since CBD enhances the singlet oxygen as a result of the protein and lipid peroxidation. In sum, research on the CBD effects must continue in order to establish how it influences the physiological roles of different cell types with clinical relevance.

## Data Availability

The data presented in this study are available on request from the corresponding author.
